# Development and validity evidence of an objective structured assessment of technical skills score for minimally invasive linear-stapled, hand-sewn intestinal anastomoses: the A-OSATS score

**DOI:** 10.1007/s00464-021-08806-2

**Published:** 2021-11-09

**Authors:** Mona W. Schmidt, Caelan M. Haney, Karl-Friedrich Kowalewski, Vasile V. Bintintan, Mohammed Abu Hilal, Alberto Arezzo, Marcus Bahra, Marc G. Besselink, Matthias Biebl, Luigi Boni, Michele Diana, Jan H. Egberts, Lars Fischer, Nader Francis, Daniel A. Hashimoto, Daniel Perez, Marlies Schijven, Moritz Schmelzle, Marek Soltes, Lee Swanstrom, Thilo Welsch, Beat P. Müller-Stich, Felix Nickel

**Affiliations:** 1 Department of General, Visceral, and Transplantation Surgery, Heidelberg University Hospital, Im Neuenheimer Feld 420, 69120 Heidelberg, Germany; 2 Department of Gynecology and Obstetrics, University Medical Centre Mainz, Langenbeckstraße 1, 55131 Mainz, Germany; 3 Department of Urology, University Hospital Leipzig, Liebigstraße 20, Haus 4, 04103 Leipzig, Germany; 4 Department of Urology and Urological Surgery, University Medical Center Mannheim, University of Heidelberg, Theodor-Kutzer-Ufer 1-3, 68167 Mannheim, Germany; 5 Department of Surgery, 1st Surgical Clinic, University of Medicine and Pharmacy, Cluj Napoca, Romania; 6 Department of Surgery, Hepatobiliary Pancreatic and Minimally Invasive Unit, Poliambulanza Foundation Hospital, Brescia, Italy; 7 Department of Surgery, University Hospital Southampton NHS Foundation Trust, Tremona Road, Southampton, UK; 8 Department of Surgical Sciences, University of Torino, Turin, Italy; 9 Department of Surgical Oncology and Robotics, Waldfriede Hospital, Berlin, Germany; 10 Department of Surgery, Cancer Center Amsterdam, Amsterdam UMC, University of Amsterdam, Amsterdam, the Netherlands; 11 Department of Surgery, Charité—Universitätsmedizin Berlin, Campus Charité Mitte/Campus Virchow Klinikum, Berlin, Germany; 12 Fondazione IRCCS-Ca`Granda - Ospedale Maggiore Policlinico di Milano, University of Milan, Milan, Italy; 13 IRCAD Research Institute Against Digestive Cancer, Strasbourg, France; 14 IHU-Strasbourg, Institute of Image-Guided Surgery, Strasbourg, France; 15 Department of General, Digestive, and Endocrine Surgery, University Hospital of Strasbourg, Strasbourg, France; 16 ICube Lab, Photonics for Health, University of Strasbourg, Strasbourg, France; 17 Department of General, Visceral, Thoracic, Transplantation and Pediatric Surgery, Kurt Semm Center for Minimally Invasive and Robotic Surgery, University Hospital Schleswig Holstein, Campus Kiel, Kiel, Germany; 18 Department of Surgery, Hospital Mittelbaden, Baden-Baden, Germany; 19 Department of Colorectal Surgery, Yeovil District Hospital Foundation Trust, Yeovil, UK; 20 Department of Surgery, Massachusetts General Hospital, Boston, MA USA; 21 Department of General, Visceral, and Thoracic Surgery, University Medical Center Hamburg-Eppendorf, Hamburg, Germany; 22 Department of Surgery, Amsterdam Gastroenterology Endocrinology Metabolism, Amsterdam UMC, University of Amsterdam, PO Box 22660, 1100 DD Amsterdam, the Netherlands; 23 1St Department of Surgery, University of Pavol Jozef Safarik, Košice, Slovakia; 24 Department of Visceral, Thoracic, and Vascular Surgery, Medical Faculty, University Hospital Carl Gustav Carus, Technische Universität Dresden, 01307 Dresden, Germany

**Keywords:** Minimally invasive surgery, OSATS, Anastomosis, Skill assessment, Delphi method

## Abstract

**Introduction:**

The aim of this study was to develop a reliable objective structured assessment of technical skills (OSATS) score for linear-stapled, hand-sewn closure of enterotomy intestinal anastomoses (A-OSATS).

**Materials and methods:**

The Delphi methodology was used to create a traditional and weighted A-OSATS score highlighting the more important steps for patient outcomes according to an international expert consensus. Minimally invasive novices, intermediates, and experts were asked to perform a minimally invasive linear-stapled intestinal anastomosis with hand-sewn closure of the enterotomy in a live animal model either laparoscopically or robot-assisted. Video recordings were scored by two blinded raters assessing intrarater and interrater reliability and discriminative abilities between novices (*n* = 8), intermediates (*n* = 24), and experts (*n* = 8).

**Results:**

The Delphi process included 18 international experts and was successfully completed after 4 rounds. A total of 4 relevant main steps as well as 15 substeps were identified and a definition of each substep was provided. A maximum of 75 points could be reached in the unweighted A-OSATS score and 170 points in the weighted A-OSATS score respectively. A total of 41 anastomoses were evaluated. Excellent intrarater (*r* = 0.807–0.988, *p* < 0.001) and interrater (intraclass correlation coefficient = 0.923–0.924, *p* < 0.001) reliability was demonstrated. Both versions of the A-OSATS correlated well with the general OSATS and discriminated between novices, intermediates, and experts defined by their OSATS global rating scale.

**Conclusion:**

With the weighted and unweighted A-OSATS score, we propose a new reliable standard to assess the creation of minimally invasive linear-stapled, hand-sewn anastomoses based on an international expert consensus. Validity evidence in live animal models is provided in this study. Future research should focus on assessing whether the weighted A-OSATS exceeds the predictive capabilities of patient outcomes of the unweighted A-OSATS and provide further validity evidence on using the score on different anastomotic techniques in humans.

**Supplementary Information:**

The online version contains supplementary material available at 10.1007/s00464-021-08806-2.

Over the past decades, minimally invasive surgery (MIS) has become the gold standard for many surgical procedures [1]. As a key component of many surgical procedures in bariatric, colorectal, and general surgery, the skill of creating minimally invasive intestinal anastomoses is of high clinical relevance [2]. Irrespective of the technique used (stapled, hand-sewn, mixed), the creation of intestinal anastomoses is considered an advanced surgical skill [3]. This results in prolonged learning curves of minimally invasive surgical procedures involving intestinal anastomoses [4–6], increasing the risk for complications. Recent studies have shown increased complication rates and decreased oncological outcomes depending on the surgeon’s level of technical skills [7, 8]. This highlights the importance of training outside of the operating room (OR) to ensure patients’ safety. While there is still a paucity of data to strengthen the role of surgical skill assessment for the certification of surgeons, training curricula with standardized assessments of surgical skills are currently incorporated in many surgical residency programs [9, 10]. Financial expenses associated with surgical skill training outside of the OR often limit the availability of training opportunities. Consequently, most surgical procedures are still taught in the OR. However, it leads to additional time spent by experienced surgeons in the OR and it increases the costs through prolonged operative times. Harrington et al. calculated an educational cost of 1457$ per laparoscopic entero-enterostomy performed by a senior surgical trainee in the OR [11], which highlights the advantages of effective technical skills training outside of the OR.

To date, there is no standard for assessing surgical competency for the minimally invasive creation of intestinal anastomoses. Most commonly, operative time is used as a competency surrogate along with monitoring learning curves of minimally invasive intestinal anastomoses and clinical outcomes such as the occurrence of a leak or obstruction [12]. Aside from outcome assessments, there is little published on procedural, technical skill assessments for minimally invasive intestinal anastomoses. The highly used Global Rating Scale of the Objective Structured Assessment of Surgical Skills (OSATS) score or the Global Operative Assessment of Laparoscopic Skills (GOALS) score are often applied to procedures where there is no procedure-specific assessment score [13–15]. Unfortunately, these general evaluations of technical surgical skills do not offer feedback about procedure-specific tasks and challenges to the trainee. As a result, many procedure-specific checklists have been created, e.g., for Nissen fundoplication [16], laparoscopic gastric bypass, and laparoscopic cholecystectomy [17–19]. One of the most noteworthy examples is the Bariatric Objective Structured Assessment of Technical Skill (BOSATS) score, which can be used for laparoscopic gastric bypass surgery. It includes multiple subscores for tasks involved in the creation of a gastric bypass including multiple scores for different types of anastomoses. However, these are often unspecific and generalized or limited to one specific technique.

Consequently, the aims of this study were the following: (1) to develop a procedure-specific assessment tool for minimally invasive linear-stapled gastrointestinal anastomoses allowing for variations in such as the use of stay sutures or suturing technique, while still providing clear definitions for each step, (2) to evaluate the influence of assigning different weights to procedural substeps according to their importance for patient outcomes based on expert judgment, (3) to gather first validity evidence on the newly developed scores for categorizing surgeons into novices, intermediates, and experts based on their performance and (4) to offer a detailed, structured, objective feedback tool for training purposes and monitoring learning curves.

## Materials and methods

The study was approved by the local ethics committee at Heidelberg University S436/2018 and by the regional council (G-161/18). A modified Delphi approach was chosen to identify and weigh key components of a minimally invasive stapled intestinal anastomosis with hand-sewn closure of the enterotomy. The Delphi survey was performed using an online survey tool (https://www.umfrageonline.com). To develop and assess the use of the anastomoses - objective structured assessment of technical skills (A-OSATS) score, the following key steps were addressed:

1. Creation of a preliminary A-OSATS score based on the available literature including definitions of substeps;
2. Delphi pre-round with an international expert panel to identify missing steps and improve/modify definitions—creation of an unweighted A-OSATS score;
3. Three Delphi iterations with an international expert panel to weigh the importance of each substep for patient outcomes;
4. Creation of a final weighted A-OSATS score based on Delphi results;
5. Gathering validity evidence in live porcine models to categorize surgeons into novices, intermediates, and experts in MIS.

### Steps 1 and 2: identifying key steps and defining critical aspects for each step by literature review and Delphi pre-round

A thorough review of the literature was performed to identify current scoring systems, which include the creation of minimally invasive anastomoses, and to identify the relevant literature on surgical techniques. Based on these results, a preliminary A-OSATS score was created. It consisted of general key steps and specific substeps. In line with prior OSATS scores, each step could be ranked on a scale from poor (1) to perfect (5) performance. Each step incorporated different aspects which should influence its rating. These aspects were included in a definition on what was expected for a poor, intermediate, and perfect performance for each substep. An international expert panel was then identified through published articles in the field, MIS expertise, and congress contributions. Clinical expertise was judged by medical licensing in a surgical field including abdominal surgery, as well as personal clinical focus on MIS (e.g., as mentioned on official hospital websites or through clinical positions such as the head of the department of MIS). Research experience was judged by the number of publications and h-Index and academic title (e.g., professor, Ph.D.). If the h-Index was low or no information could be found, the published papers were screened for papers focusing on MIS, anastomosis, surgical education and technique. An overview of these qualifications can be found in the Supplementary Material 1. In a pre-Delphi round, all experts received the preliminary A-OSATS score and were asked to decide on the inclusion/exclusion of each step, propose new steps with definitions, and/or provide new/modified aspects which should be incorporated in the definitions of each step. The aim of this pre-Delphi round was to defining the final inclusion of steps and definitions before rating their importance in the following Delphi rounds. All feedback was critically reviewed by the main authors. If indicated, substeps were either merged together, deleted, or modified according to the feedback received based on a consensus of the main authors. This step resulted in the unweighted A-OSATS score.

### Step 3: weighing of steps according to clinical relevance on patient outcomes

**Fig. 1:**
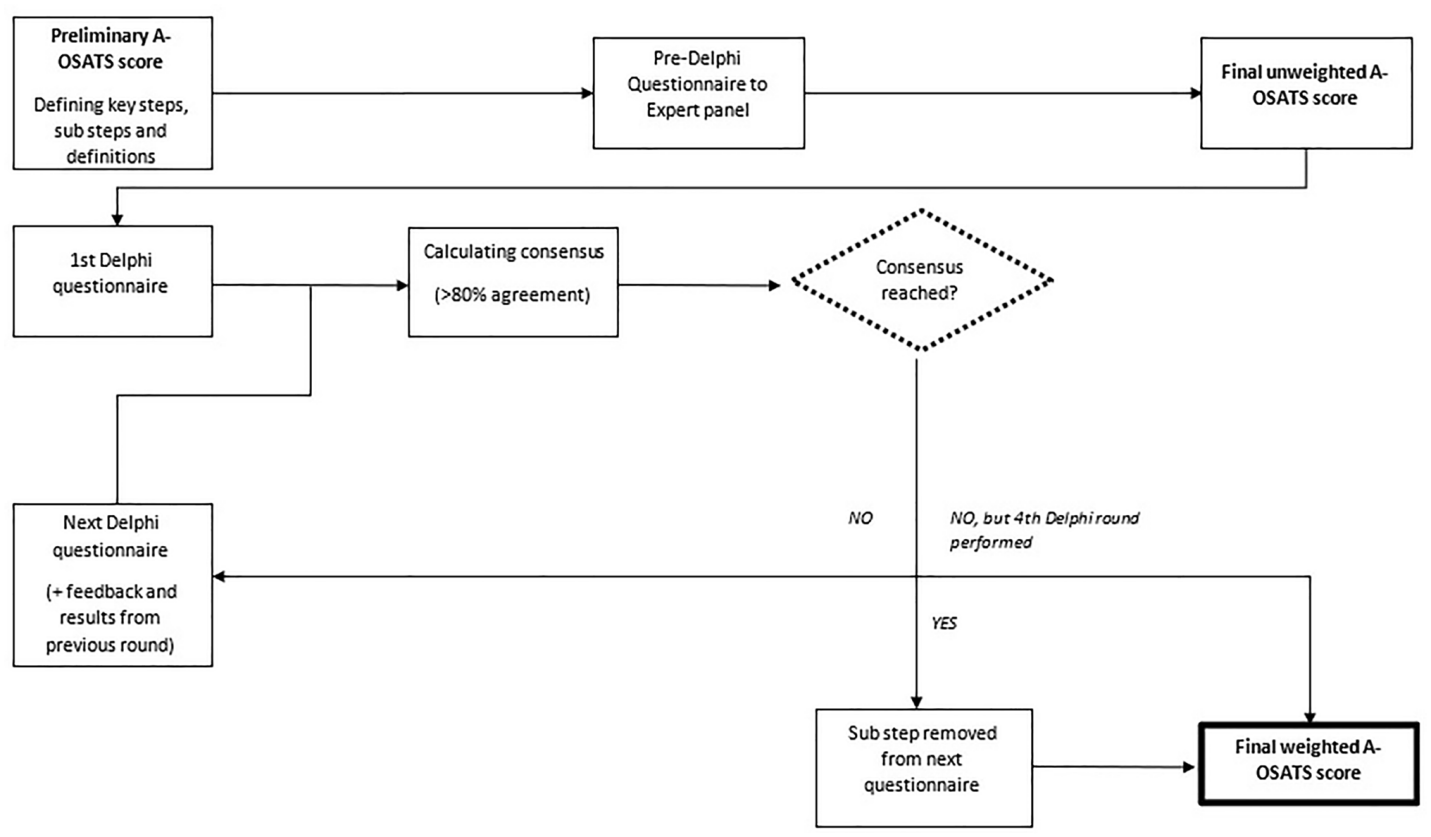
Flowchart of modified Delphi process

The final unweighted A-OSATS score was sent out to the international expert panel to determine the relevance of each substep for patient outcomes. An example of the questionnaire can be found in Supplementary Material 2. The relevance for patient outcome was based on the experts` opinions. Three categories for determining the rank of importance were put forward, namely minor, intermediate, and major importance for patient outcomes. Experts were asked to assign each step to one category and comment on their choice. Comments and results including the trend from the previous round were then offered anonymously to the expert panel for reconsideration in the following rounds. All substeps reaching a predefined level of consensus were discarded in the following rounds. Consensus was defined as > 80% agreement on one category. If no consensus was reached after round 4, the category was assigned according to the majority of votes. Should two categories have equal votes, the trend during the three rounds was used to decide between those categories. Two reminders were sent per mail before closing each round. A flowchart of the Delphi methodology used can be seen in Fig. 1.

### Step 4: creation of final A-OSATS score

In the next step, the weights were incorporated into the unweighted A-OSATS score. The value of each step which was assigned to the category “major importance for patient outcome”, was multiplied by three. Each value of the category “intermediate importance” was multiplied by two and each category of “minor importance” was multiplied by one. As a result, the scale of a step with major importance now ranged from 3 to 15 (in steps of 3) as opposed to a scale of 1 to 5 for steps with minor importance (Table 1).

**Table 1:** Weighing system based on the importance of steps for patient outcomes as compared to conventional objective structured assessment of technical skills (OSATS) rating

	“Poor” performance		“Intermediate” performance		“Perfect” performance
*Traditional/Unweighted (A-)OSATS rating*					
All steps	1	2	3	4	5
*Weighted A-OSATS rating*					
Steps with minor clinical importance*	1	2	3	4	5
Steps with intermediate clinical importance*	2	4	6	8	10
Steps with major clinical importance*	3	6	9	12	15

*OSATS* objective structured assessment of technical skills, *A-OSATS* objective structured assessment of technical skills score for linear-stapled, hand-sewn closure of enterotomy intestinal anastomoses

*Importance for patient outcome

### Step 5: gathering validity evidence for the final A-OSATS scores

This study was conducted in the training center for Minimally Invasive Surgery of the Department of General, Visceral, and Transplantation Surgery at Heidelberg University Hospital, Germany. Written informed consent was obtained from each participant after providing information on type, extent, and value of this study. Participants had the option to withdraw consent at any time and without reasoning. All participants were surgeons, residents, or medical students at Heidelberg University Hospital.

To gather validity evidence for the use of the unweighted A-OSATS score and the weighted A-OSATS score to categorize surgical skill, participants with different experience levels in MIS were asked to perform a laparoscopic or robot-assisted anastomosis in a live porcine model, after watching a short introduction video. Participants were allowed to ask questions prior to performing the procedure. All participants were grouped according to surgical skills demonstrated during the study, as assessed by the OSATS GRS score. As there were no specifically defined cut-off scores to categorize trainees according to the OSATS GRS, trainees with OSATS GRS scores ≤ 18 were considered novices, between 19 and 27 intermediates, and ≥ 28 experts based on tentative cut-off scores in previously published studies [17, 20]. Additionally, the study population was pragmatically divided into experts, intermediates, and novices based on the number of prior minimally invasive anastomoses performed. Experts were defined as having performed more than 10 minimally invasive anastomoses, intermediates between 1 and 10 minimally invasive anastomoses, and novices none. One trained tutor assisted each participant during the procedure by guiding the camera and performing helping maneuvers only when instructed by the operating participant. All procedures were recorded and the videos were evaluated by two blinded raters using the weighted and unweighted A-OSATS score. Both weighted and unweighted A-OSATS scores were assessed to identify possible positive or negative consequences of weighting the substeps differently. Furthermore, the same videos were rated by the blinded raters using the standard OSATS GRS to categorize the participants into novice/intermediates and experts [15]. Each video was evaluated twice by each blinded rater in a random fashion. Time was recorded with predefined start/stop criteria (Start: 5 s before the first stitch; Stop: End of the final knot/end of anastomotic inspection if performed).

### Statistical evaluation

All statistical tests were performed using SPSS (Version 27, IBM SPSS Inc., Chicago, Illinois, USA). Graphs were created using STATA (Version 16, StataCorp LLC, College Station, Texas, USA). All tests were two-sided and a *p* value of < 0.05 was regarded as statistically significant. Continuous data were reported as means and standard deviation, whereas ordinal data were reported as median and interquartile ranges. Interrater reliability was assessed with the intraclass correlation coefficient (ICC) type 2,1 according to Shrout and Fleiss [21]. The second round of ratings was used to assess interrater reliability, and ICCs of less than 0.5, between 0.5 and 0.75, between 0.75 and 0.9, and greater than 0.9 were regarded as poor, moderate, good, and excellent reliability, respectively [22]. Intrarater reliability and correlation of (weighted) A-OSATS with OSATS was evaluated using the Spearman correlation. Correlation coefficients of 0–0.3, 0.3–0.5, 0.5–0.7, 0.7–0.9, and > 0.9 were regarded as negligible, low, moderate, high, and very high correlation, respectively [23]. The Kruskal–Wallis test was used to assess a difference in (weighted) A-OSATS between three groups of different experience levels (novices, intermediates, experts), with Dunn’s test used for post hoc analysis. Only the second round of ratings from rater 1 was used for all further analyses. This approach was deemed appropriate based on an excellent interrater reliability.

## Results

### Modified Delphi survey: creation of (weighted) A-OSATS score

Four key steps (intestinal placement, creation of enterotomies, stapling, and closure of enterotomy) were identified along with 16 substeps in the preliminary A-OSATS score. Important factors to assess within each substep were identified and included in the proposed definitions of each substep. Nineteen international MIS experts from 8 countries participated in this study. All experts participated in more than one round, and 9 experts completed all rounds. For participation rates of each round, please see Table 2. Participants of all Delphi rounds always answered the complete questionnaire. The preliminary A-OSATS score was adjusted based on the feedback received from the expert panel in the pre-Delphi round. The final unweighted A-OSATS score consisted of the same four key steps as the preliminary A-OSATS and a total of 15 substeps (Table 3).

**Table 2:** Participants and level of consensus during the Delphi survey

	Pre-Delphi Round	Round 1	Round 2	Round 3	Round 4
Total number of participants	19	18	18	18	11
Number of sub steps where consensus was reached*	N/A	0/15	2/15	8/15	9/15

*Sub steps with consensus > 80% agreement out of 15 sub steps

**Table 3:** Weighted and unweighted objective structured assessment of technical skills score for minimally invasive linear-stapled, hand-sewn closure of enterotomy intestinal anastomoses (A-OSATS)

Bowel placement and setup			
Choice and preparation of bowel segments for anastomosis	- Bowel segments not tension free - Diameter of bowel segment too small for stapler	- Bowel segments checked, diameter most likely big enough for stapler and segments most likely tension free	Bowel segments checked, diameter big enough for stapler and tension free
Points	1	3	5
Points weighted	3	9	15
Bowel positioning	- Bad bowel positioning (with or without stay sutures) resulting in great difficulties	- Inadequate bowel positioning (with or without stay sutures) resulting in minor difficulties	- Entire procedure completed without problems (with or without stay sutures)
Points	1	3	5
Points weighted	2	6	10

Creation of enterotomies			
Handling of tissue during creation of enterotomies	- Excessive tissue damage - Inappropriate instrument is used to facilitate creation of enterotomies	- Little tissue damage - Appropriate instrument used to facilitate creation of enterotomies, but insecure handling	- Tissue is not damaged - Appropriate instrument used to facilitate creation of enterotomies
Points	1	3	5
Points weighted	3	9	15
Quality of enterotomies	- Enterotomies do not incorporate all layers of intestine - Enterotomies badly frayed and excessively larger or smaller than necessary	- Enterotomies incorporate all layers of intestine - Enterotomies slightly frayed and either slightly loo large or too small	- Enterotomies incorporate all layers of intestine - Sides of enterotomies not frayed and of ideal size
Points	1	3	5
Points weighted	2	6	10
Location of enterotomies	- Enterotomies in inappropriate location (e.g., too close to mesentery or too close to end of bowel segment)	- Enterotomies placed in a possible location but there is an increased risk of tension or difficult suturing after stapling	- Enterotomies placed appropriately and without risk of tension or difficult suturing after stapling
Points	1	3	5
Points weighted	3	9	15

Stapling			
Handling of tissue during insertion/extraction of stapler	- Excessive tissue tears - Inappropriate instrument is used to facilitate entry of stapler jaws	- Small tissue tears - Appropriate instrument used to facilitate entry of stapler jaws, but mucosa grabbed	- Tissue is not damaged - Appropriate instrument used to facilitate entry of stapler jaws
Points	1	3	5
Points weighted	2	6	10
Final placement of stapler before firing	- Stapler placed into wrong part of intestine - Tissue placed asymmetrically or overlaps inside stapler	- Tissue slightly asymmetrical inside stapler	- Tissue placed symmetrically and does not overlap inside stapler
Points	1	3	5
Points weighted	2	6	10
Inspection of stapler placement and compression time	- Placement of stapler not checked - Compression time as advised by stapler manufacturer not maintained	- Not all parts of anastomosis adequately inspected - Compression time as advised by stapler manufacturer not maintained entirely	- Thorough inspection of all parts of anastomosis - Compression time maintained as advised by stapler manufacturer
Points	1	3	5
Points weighted	2	6	10
Final shape of enterotomy	- Enterotomy excessively large, frayed, or asymmetrical	- Enterotomy slightly large, frayed, or asymmetrical	- Enterotomy small, symmetrical, and well-shaped
Points	1	3	5
Points weighted	2	6	10
Hemostatic checking of stapler line	- Possibility of bleeding not checked or checked but not handled appropriately	- Possibility of bleeding not checked sufficiently, but obvious bleeding handled appropriately	- Thorough inspection of stapler line for bleeding and bleeding handled appropriately
Points	1	3	5
Points weighted	2	6	10

Closure of enterotomy			
Closure of first transition zone between stapler line and enterotomy	- Transition zone not closed properly, excessive tissue damage - Thread rarely lands in good position - Insufficient knot tying and excessively large/small stitch distance	- Transition zone closed with minor inaccuracies, hardly any tissue damage - Thread usually lands in good position - Final knots and stitch distance are mostly secure and appropriate	- Transition zone closed up appropriately, no tissue damage - Thread always lands in a good position - Knots all tied well, and stitch distance is always appropriate
Points	1	3	5
Points weighted	2	6	10
Closure of middle part of enterotomy	- Middle part not closed properly, excessive tissue damage - Thread rarely lands in good position - Insufficient knot tying and excessively large/small stitch distance	- Middle zone closed with minor inaccuracies, hardly any tissue damage - Thread usually lands in good position - Final knots and stitch distance are mostly secure and appropriate	- Middle zone closed up appropriately, no tissue damage - Thread always lands in a good position - Knots all tied well, and stitch distance is always appropriate
Points	1	3	5
Points weighted	2	6	10
Closure of second transition zone between stapler line and enterotomy	- Transition zone not closed properly, excessive tissue damage - Thread rarely lands in good position - Insufficient knot tying and excessively large/small stitch distance	- Transition zone closed with minor inaccuracies, hardly any tissue damage - Thread usually lands in good position - Final knots and stitch distance are mostly secure and appropriate	- Transition zone closed up appropriately, no tissue damage - Thread always lands in a good position - Knots all tied well, and stitch distance is always appropriate
Points	1	3	5
Points weighted	2	6	10
Suturing technique	- Inappropriate instruments used - Multiple attempts for stitches constantly needed - Not all necessary layers of tissue included in stitches or included asymmetrically	- Appropriate instruments used, mucosa grabbed occasionally - Multiple attempts needed for stitches - All necessary layers of tissue included symmetrically	- Appropriate instruments used without grabbing mucosa - Only one attempt needed for each stitch - All necessary layers of tissue included symmetrically
Points	1	3	5
Points weighted	3	9	15
Inspection of suture line and additional placement of stitches if necessary	- Suture line not inspected - Additional stitches or second suture line not placed even though necessary - Loose knots with a high chance of opening are not replaced - Serosal damage not repaired	- Not all parts of suture line inspected - Additional stiches or second suture line placed if necessary - Obviously loose knots with a high chance of opening are replaced - Serosal damage repaired	- All parts inspected thoroughly - If additional stitches or second suture line are needed, these are placed in appropriate position - All knots tied securely - Serosal damage repaired
Points	1	3	5
Points weighted	2	6	10

During four rounds of the modified Delphi survey, the international expert panel then evaluated the importance of each substep for patient outcomes. A consensus was reached for 9 substeps after round 4 (Table 2). For the remaining 6 substeps, the final category was determined based on majority votes. Four substeps reached a majority with more than 72% and two substeps with more than 63%. These majorities fell in line with the trend observed during the previous rounds. The final weighted A-OSATS score including substeps, definitions, and weights are displayed in Table 3. A total of 75/170 points (unweighted/weighted) can be reached during the creation of a minimally invasive linear-stapled intestinal anastomosis with hand-sewn closure of the enterotomy.

### Validity evidence for the A-OSATS score

40 participants were recruited to present validity evidence for the use of the A-OSATS score to classify a surgeon into novice/intermediate or expert based on their performance. Demographics and experience of all participants can be seen in Table 4. A total of 41 anastomoses were performed, 27 of which were laparoscopic and 14 robotically assisted. All participants completed the study.

**Table 4:** Demographics of study participants

	According to number of anastomoses performed			According to OSATS score		
	Novices *n* = 20	Intermediates *n* = 13	Experts *n* = 3	Novices *n* = 8	Intermediates *n* = 24	Experts *n* = 8
Age (Mean ± SD)	30.5 ± 6.1	29.3 ± 5.8	43.3 ± 1.2	27.4 ± 3.3	30.6 ± 7.0	36.4 ± 6.0
Years of clinical experience [Median (IQR)]	2 (1–6)	1 (0–4)	44 (42–44)	1 (1–4)	1 (0–5)	9.5 (5–13.5)
Time taken for anastomosis (min)* (Mean ± SD)	40.9 ± 17.0	33.6 ± 12.3	24.3 ± 15.0	47.8 ± 17.8	36.1 ± 15.1	29.5 ± 13.3
OSATS Score* (Mean ± SD)	22.2 ± 4.9	23.2 ± 4.2	32.5 ± 4.4*	16.0 ± 2.0	23.2 ± 2.4	31 ± 3.0*

*OSATS* objective structured assessment of technical skills

*One Expert performed both a laparoscopic and a robot-assisted anastomosis, thus the analyzed outcome data includes 4/9 anastomoses respectively in the Expert groups

Both raters demonstrated a high intrarater reliability for the unweighted and weighted A-OSATS (Table 5). In addition, an excellent interrater reliability was seen for both A-OSATS (unweighted A-OSATS: ICC = 0.923, *p* < 0.001; weighted A-OSATS: ICC = 0.924, *p* < 0.001).

**Table 5:** Intrarater reliability

	A-OSATS	Weighted A-OSATS
Rater 1	*r* = 0.807**	*r* = 0.790**
Rater 2	*r* = 0.988**	*r* = 0.985**

*A-OSATS = objective structured assessment of technical skills score for linear-stapled, hand-sewn closure of enterotomy intestinal anastomoses

**Fig. 2:**
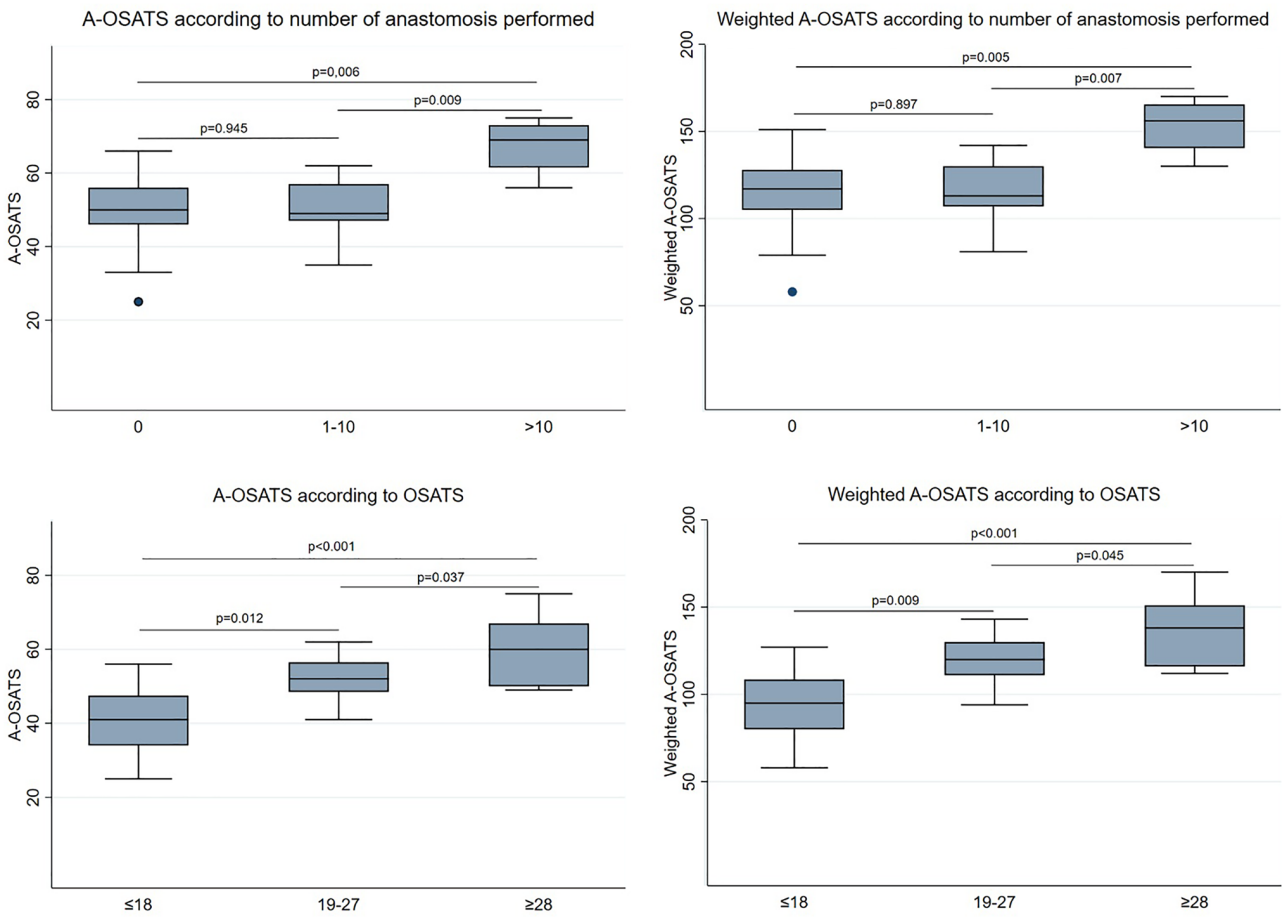
Unweighted and weighted objective structured assessment of technical skills score for minimally invasive linear-stapled, hand-sewn closure of enterotomy intestinal anastomoses (A-OSATS) according to different levels of experience

Both scores correlated highly with the non-specific OSATS (unweighted AOSATS: *r* = 0.810, *p* < 0.001; weighted OSATS: *r* = 0.827, *p* < 0.001) and with each other (*r* = 0.996, *p* < 0.001). In general, both unweighted and weighted A-OSATS could differentiate between three different levels (novices, intermediates, experts) of experience (*p* < 0.05). However, when categorizing participants according to the number of anastomoses performed, neither unweighted nor weighted A-OSATS could differentiate between novices and intermediates (Fig. 2).

## Discussion

This study presents the newly developed assessment scores for minimally invasive linear-stapled gastrointestinal anastomoses—namely weighted and unweighted A-OSATS scores, as well as first validity evidence for its use to rate a surgeon`s performance in a porcine model. The scores are based on an international expert consensus to determine relevant steps and their importance for patient outcomes. In a porcine model, both scores demonstrate excellent interrater and intrarater reliability, as well as discriminative capabilities to differentiate between novices, intermediates, and experts when classified according to their OSATS GRS level of skill. When classified according to their previous experience in minimally invasive anastomoses, the score was able to discriminate between experts and novices/intermediates but no longer between intermediates and novices.

The adequate assessment of surgical skills is of utmost importance to ensure patient safety and improve clinical outcomes. As recent studies have shown, technical surgical skills correlate with patient outcomes [7, 8]. Objective assessment methods cannot only provide the necessary evaluation of surgical skills, but they can simultaneously provide trainees with feedback regarding their own strengths and weaknesses and they can be used to evaluate the learning curve [24]. As a result, objective assessment scores have the potential to not only function as credentialing tools, but also to enhance both surgical training outside and inside of the operating room. However, in order to ensure an appropriate use, assessment scores need to be accurate, reliable, and comparable regardless of the user. Before using a scoring tool validity evidence needs to be presented, assuring that the score adequately represents the construct it aims to measure [25]. Currently accepted validity frameworks by Messick [26] and Kane [27] describe the overarching framework of construct validity, which is supported by various aspects of evidence. While a tool itself can never be validated, validity evidence to support its interpretation can be gathered. A practical guide to gather validity evidence based on Kane`s framework for OSATS scores has been presented by Cook et al. [28]. In line with the presented suggestions by Cook et al., we provide first validity evidence from various key elements including “scoring inference evidence” by providing descriptions/definitions of each item and demonstrating its potential use by video ratings thus showing the translation of a performance into a score. Furthermore, we provide “generalization evidence” by high inter- and intrarater reliability. Finally, we provide “extrapolation evidence” by ensuring that each item adequately represents an important skill aspect through the creation of the A-OSATS score based on the opinions of known-experts in the field of minimally invasive abdominal surgery and its correlation with independent ratings of the OSATS GRS scores as well as its discriminative abilities between novice, intermediate, and expert performances.

Many currently existing assessment scores include crude definitions of each step [13–15]. Therefore, it remains most often unclear as to which specific aspects should be evaluated and how. While training raters of clinical studies might lead to adequate interrater reliability within the study, comparability across studies can be affected. Additionally, inconsistent ratings would also prohibit the use of objective assessment scores as credentialing tools. Consequently, the (weighted) A-OSATS score includes definitions of each aspect which is relevant to the step, aiming to clarify and facilitate consistent ratings irrespective of the user. In addition, clear definitions allow trainees to use the A-OSATS score not only as a tool to measure their learning curve, but also as an educational tool to identify crucial steps for the correct execution of the procedure.

One of the major advantages of the A-OSATS score lies in the flexibility to incorporate individual adjustments according to the surgeons` preferences. While it is designed for the creation of a linear-stapled, hand-sewn anastomosis, it allows for deviations in the specific technique such as the use of stay sutures or continuous versus interrupted sutures. In addition, it can be used for laparoscopic and robot-assisted surgeries, as demonstrated in this study. This ensures a broad comparability and use of the A-OSATS score across different hospitals or countries.

Composite assessment scores do not often adequately reflect the concept that they are trying to measure, if steps with different relevance are incorporated as equivalent. As a result, the weighted A-OSATS score aims to give a greater importance to steps that, if not performed correctly, are more likely to affect patient outcomes, based on the judgment of international experts in MIS. In this study, the weighted and unweighted A-OSATS scores showed similar results with regards to interrater and intrarater reliability, as well as discriminative abilities. Consequently, future studies should aim to assess whether the weighted A-OSATS score reflects patient outcomes better than the unweighted A-OSATS score as hypothesized.

The Delphi method as a survey process is characterized by multiple survey iterations based on statistical group response evaluations and controlled feedback allowing for the reevaluation of one´s opinion based on responses given by other members of the expert panel while ensuring anonymity [29]. Due to these qualities, the Delphi method and its modified forms are widely established and are commonly used to create scoring systems based on experts’ opinions in the medical field [30–32]. However, the definition of when a consensus is reached often varies widely and it may include a number of rounds or a specific percentage of agreement to be reached. In line with commonly used definitions of consensus, a percentage of agreement greater than 80% was used in this study [33]. While the Delphi process can be continued until full consensus or stability is reached, a limit of one pre-Delphi questionnaire and four Delphi iterations was set to prevent participant fatigue and this lies within the upper range of the suggested number of iterations [34, 35]. With 18 panel members (except for round 4 with 11 participants), this survey excels the often suggested range of 8 to 15 panel members for homogeneous groups [36]. Even though a drop in the participation rate was observed from round 3 to round 4, all trends in changes from round to round have been stable as compared to previous rounds. Consequently, no misleading results due to this change in panel members are expected. Similarly, while six substeps did not meet the predefined criterium of > 80% agreement by round four, their relatively high agreement rates of > 63% and > 72% and matching observable trends during the survey process suggest a reasonable final result of this process, reflecting the panel’s opinion.

## Limitations

There are some limitations to consider when interpreting the results of this study. For one, neither the weighted nor the unweighted score could differentiate between novices and intermediates according to the number of prior anastomoses. This could well be due to the inclusion of several participants as intermediates with a very limited prior experience in suturing intestinal anastomoses. It is likely that these participants had not yet overcome the initial learning curve for gastrointestinal anastomoses and were subsequently comparable to novices with generally more experience in MIS and who had not yet created an anastomosis. This theory is supported by the clear distinction of novices/intermediates and experts according to the number of anastomoses performed, despite the relatively small sample size. In addition, when categorizing each participant according to the demonstrated surgical skill based on the commonly used OSATS score, both the weighted and unweighted A-OSATS could clearly distinguish between novices, intermediates, and experts. While we believe the inclusion of robot-assisted and laparoscopic anastomoses increases the generalizability and thus use of our results, there is a chance of creating heterogenous data influencing the results. Thus, a comparison between groups only performing laparoscopic anastomoses can be found in Supplementary Material 3. Due to the small number per group in robot-assisted surgery, no separate analysis was performed for robot-assisted surgery. The results in laparoscopic surgery only, are almost identical to the here presented combined robot-assisted/laparoscopic surgery analysis, thus, supporting the conclusions in this manuscript. While the A-OSATS score allows the use of variations in surgical techniques, it is limited to linear-stapled, hand-sewn anastomoses. Nevertheless, on the basis of the A-OSATS score, new assessment methods for completely stapled or completely hand-sewn anastomoses can be created since most relevant aspects have already been identified and defined within the A-OSATS. Both versions of the A-OSATS score were evaluated in live animal models. Even though no conclusions on the use of the A-OSATS for intraoperative assessments can be drawn, the live animal model simulates a very realistic intraoperative scenario, and the transferability of basic technical skills from a simulated setting to the OR has already been proven in various settings [37]. In addition, as mentioned above, the weighted A-OSATS score has been created to predict patient outcomes more adequately. However, this needs to be evaluated in future studies, as no long-term outcome data were collected during this study.

## Conclusion

With the creation of the A-OSATS score, a new learning and assessment tool is proposed to evaluate technical surgical skills during the creation of laparoscopic and robot-assisted intestinal anastomoses. The weighted version of the A-OSATS score incorporates the relative importance of each step for patient outcomes according to an international expert consensus. Both versions demonstrated excellent intrarater and interrater reliability, as well as discriminative capabilities for surgical expertise in live animal models. Future studies are necessary to evaluate their use on human patients as well as the predictability of patient outcomes using both versions of the A-OSATS score.

## Supplementary Information

Below is the link to the electronic supplementary material.

Supplementary file1 (DOCX 950 KB)
