# Supplementary material for: Development and validity evidence of an objective structured assessment of technical skills score for minimally invasive linear-stapled, hand-sewn intestinal anastomoses: the A-OSATS score
Source: Surg Endosc. 2021 Nov 9;36(6):4529–41. doi: 10.1007/s00464-021-08806-2 (PMC9085690; doi:10.1007/s00464-021-08806-2)

**Supplementary material**

**S1 Table of expert panel members` qualifications**

| Expert | Country | Medical specialty | Clinical focus on MIS | Research in the field of MIS | Highest academic title | H-Index | Number of citations |  |
| --- | --- | --- | --- | --- | --- | --- | --- | --- |
| 1 | Romania | Surgery | Yes | Yes | MD, Ph.D | 13 | 568 |  |
| 2 | Italy/UK | Surgery | Yes | Yes | Professor | 47 | 9018 |  |
| 3 | Italy | General surgery | Yes | Yes | Professor | 44 | 6244 |  |
| 4 | Germany | Surgery | Yes | Yes | Professor | 37 | 5478 |  |
| 5 | Netherlands | Surgery | Yes | Yes | Professor | 76 | 24054 |  |
| 6 | Germany | Surgery, Visceral surgery | Yes | Yes | Professor | 25 | 1903 |  |
| 7 | Italy | Surgery | Yes | Yes | Professor | 40 | 5833 |  |
| 8 | France | General surgery | Yes | Yes | MD, Ph.D | 31 | 3203 |  |
| 9 | Germany | Surgery | Yes | Yes | Professor | - | - |  |
| 10 | Germany | General and visceral surgery | Yes | Yes | Professor | - | - |  |
| 11 | UK | Surgery | Yes | Yes | Professor | 29 | 4035 |  |
| 12 | USA | General surgery | Yes | Yes | MD, MS | 16 | 984 |  |
| 14 | Germany | General, visceral and thoracic surgery | Yes | Yes | Professor | 22 | 1976 |  |
| 15 | Netherlands | Surgery | Yes | Yes | Professor | 38 | 5363 |  |
| 15 | Germany | Visceral surgery | Yes | Yes | Professor | 20 | 1664 |  |
| 16 | Slovakia | Surgery | Yes | Yes | Associate Professor | 8 | 376 |  |
| 17 | France | General surgery | Yes | Yes | Professor | 69 | 16200 |  |
| 18 | Germany | Surgery | Yes | Yes | Professor | - | - |  |
| 19 | Germany | General and visceral surgery | Yes | Yes | Professor | 39 | 4954 |  |

- Numbers not available on researchgate,com or googlescholar.com (accessed July 2021)

MIS = minimally invasive surgery

**S2 Example of the type of question and feedback included in the Delphi questionnaire (Delphi round 2)**

Figure 1: Example of Delphi questionnaire round 2


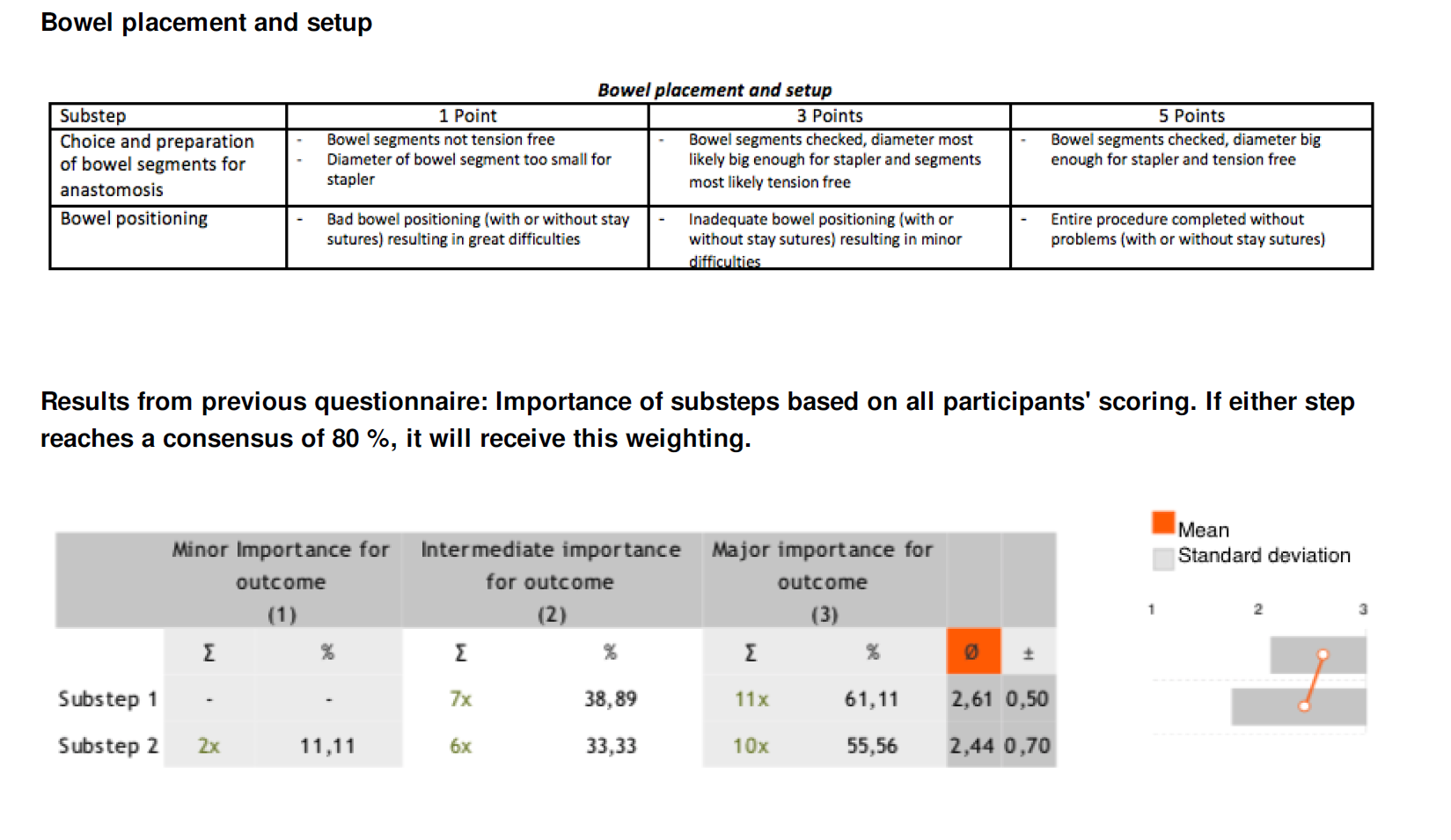

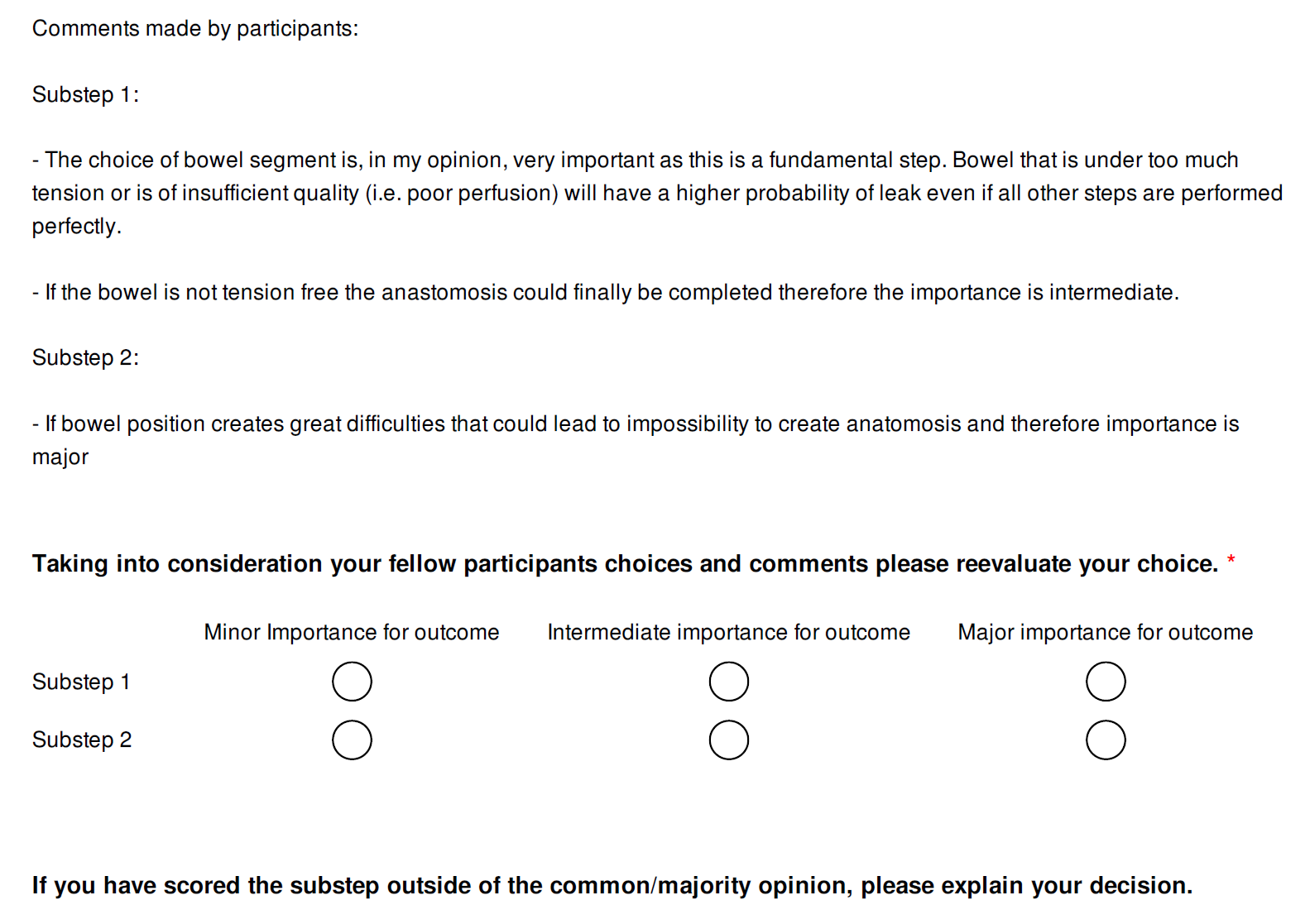


**S3 Separate analysis for laparoscopic surgery only**

Figure 1: Comparison of A-OSATS scores by number of anastomoses performed


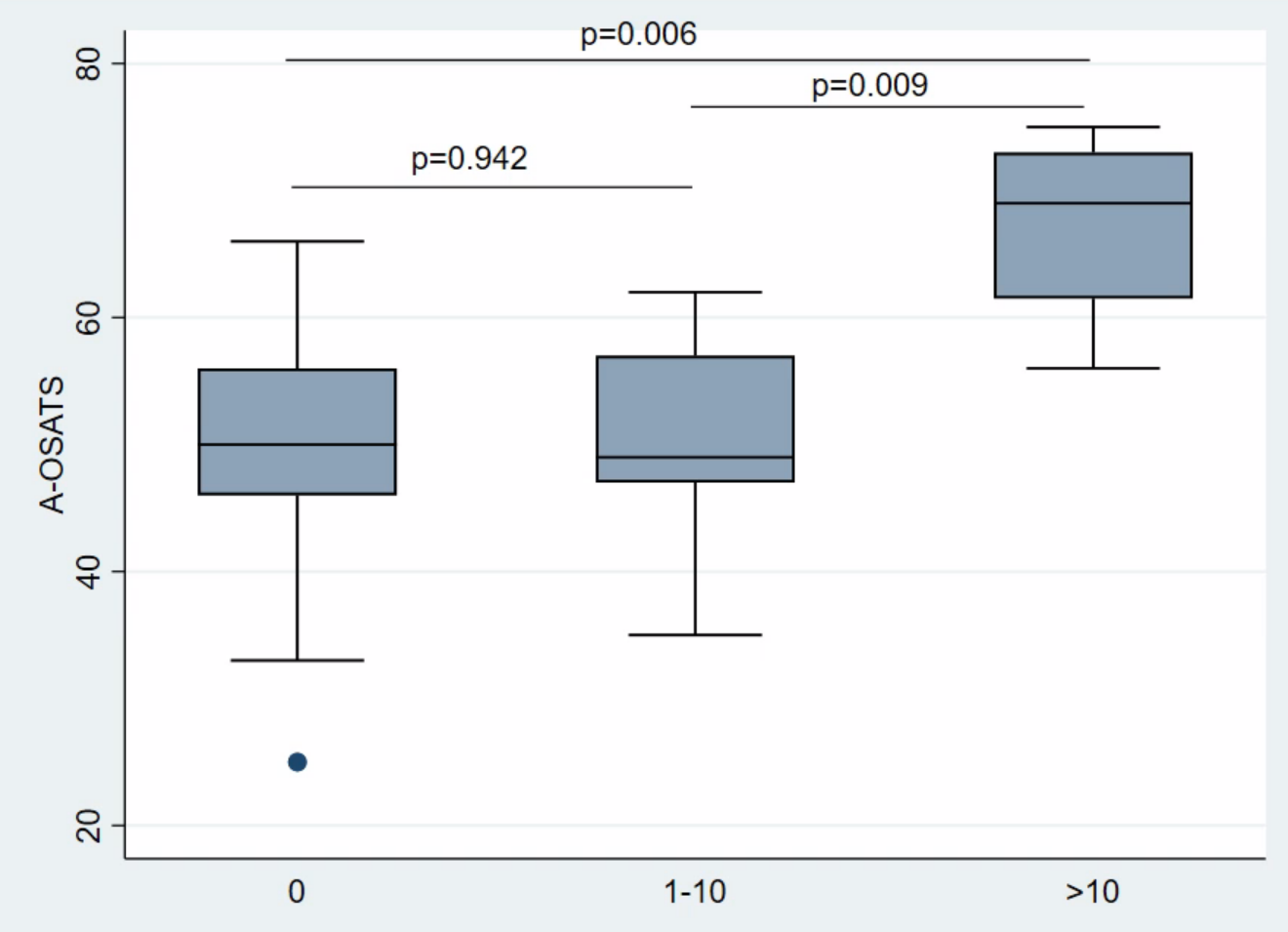


Figure 2: Comparison of weighted A-OSATS scores by number of anastomoses performed


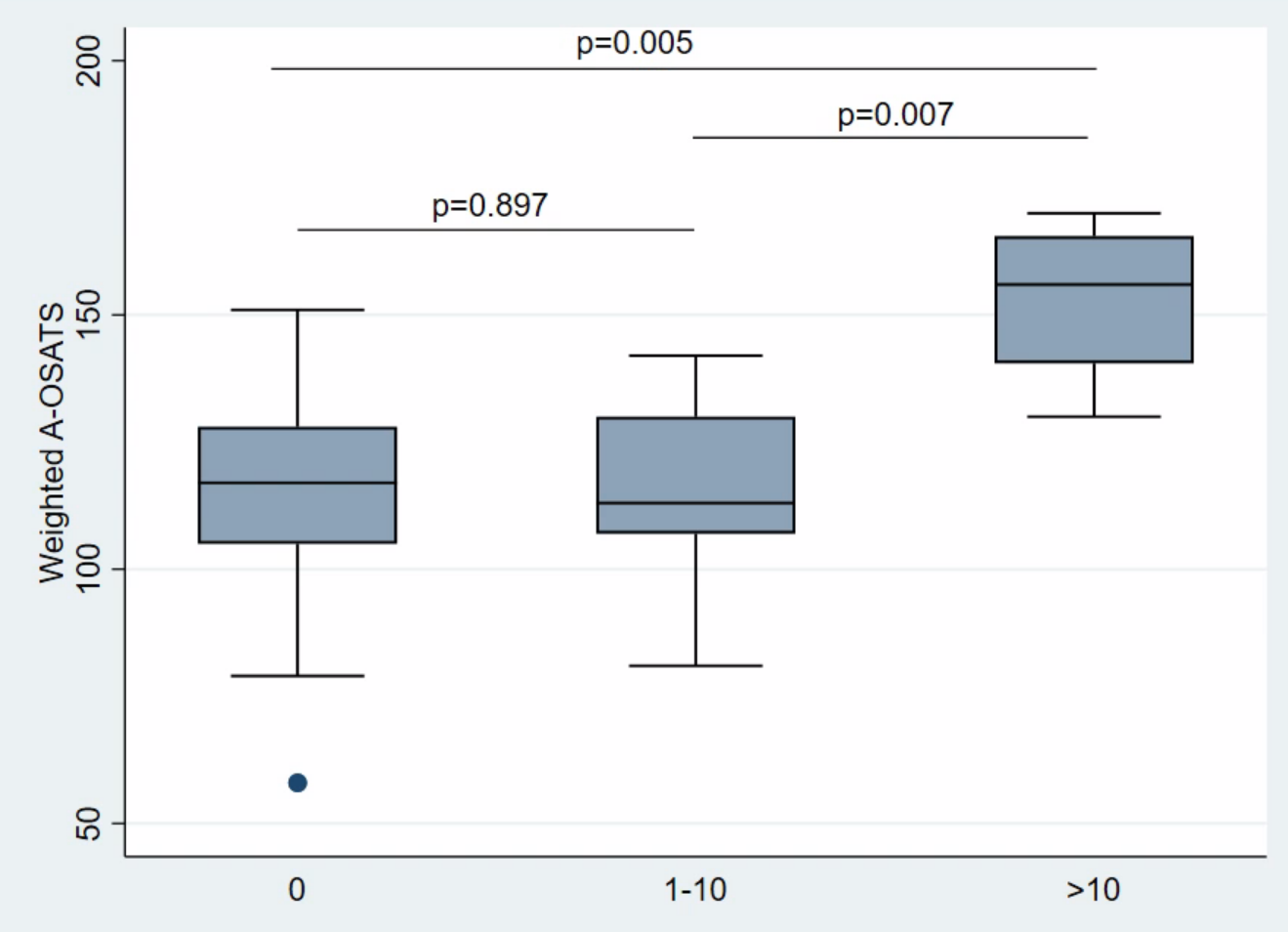


Figure 3: Comparison of A-OSATS scores by OSATS GRS scores


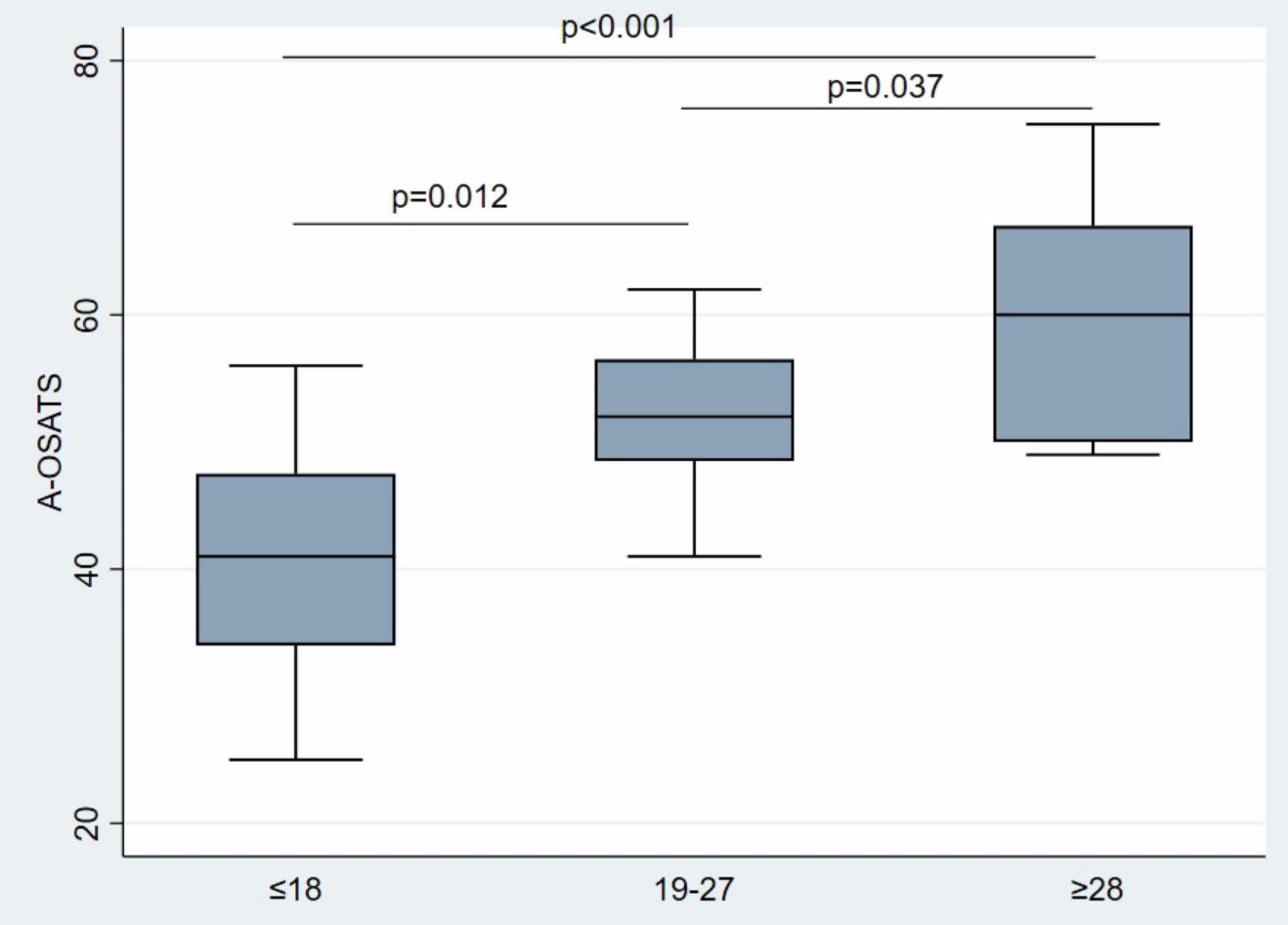


Figure 4: Comparison of weighted A-OSATS scores by OSATS GRS scores


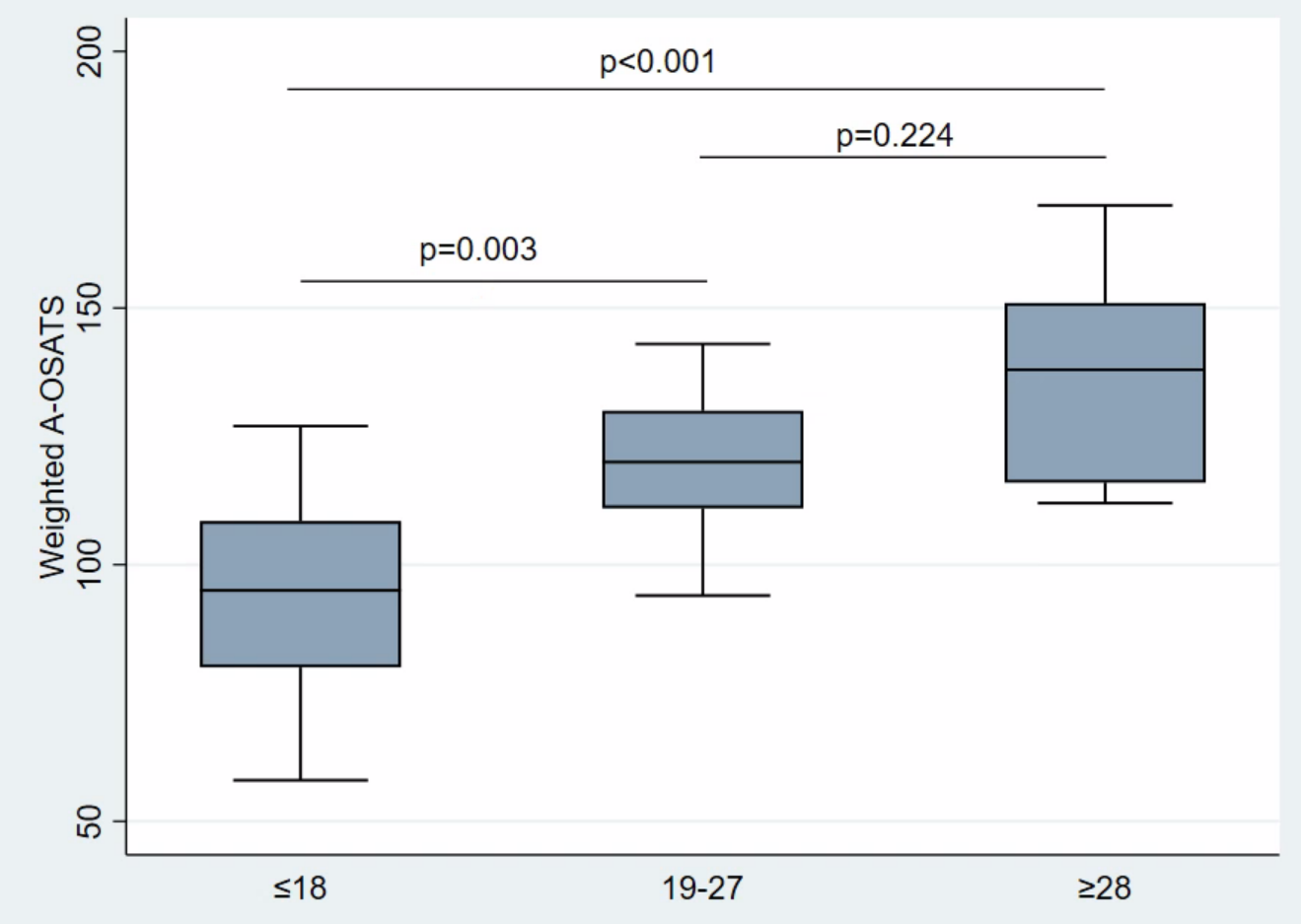

Supplement: Supplementary file 1 — Supplementary file1 (DOCX 950 KB) [file 464_2021_8806_MOESM1_ESM.docx]
